# Nurturing Opportunities: Assessing the Challenges and Engagement of Young Persons With Disabilities in Ghana′s Agribusiness Sector

**DOI:** 10.1155/oti/2851812

**Published:** 2026-07-16

**Authors:** Dan F. Acquaye, Juliana Asante-Dartey, Abraham Sarfo, Georgina Ama Gyan, Sylvester Kyei-Gyamfi, Nii Wellington, Frank Kyei-Arthur

**Affiliations:** ^1^ General Administration, Agri-Impact Group Limited, Accra, Greater Accra Region, Ghana; ^2^ Agribusiness and Private Sector Unit, Agri-Impact Group Limited, Accra, Greater Accra Region, Ghana; ^3^ Gender, Youth, Social Inclusion Department, Agri-Impact Group Limited, Accra, Greater Accra Region, Ghana; ^4^ Department of Children, Ministry of Gender, Children and Social Protection, Accra, Greater Accra Region, Ghana; ^5^ Research, Capacity-Building, Monitoring Division, Development Education Centre, Accra, Greater Accra Region, Ghana; ^6^ Department of Environment and Public Health, University of Environment and Sustainable Development, Somanya, Eastern Region, Ghana, uesd.edu.gh

**Keywords:** agribusiness, disability and employment, Ghana, inclusive capability theory, structural exclusion, young persons with disabilities

## Abstract

This study investigates the engagement of young persons with disabilities (YPWDs) in agribusiness in Ghana, where participation remains limited despite commitments to inclusive development. Using a mixed‐methods design, the research incorporates quantitative survey data from 169 respondents (97 females and 72 males) and qualitative insights from in‐depth interviews and focus group discussions. The findings revealed that 52.8% of currently employed YPWDs were engaged in agribusiness. These were primarily older female youth with limited formal education. Participants identified seven barriers: physical inaccessibility, stigma and discrimination, lack of land, lack of financial support, limited training opportunities, inadequate knowledge, and general disinterest in the sector. Guided by inclusive capability theory (ICT), the analysis shows how social norms, institutional gaps, and environmental factors restrict YPWDs′ ability to transform interest into meaningful agricultural participation. While the findings align with global evidence on youth disengagement from agriculture, this study emphasizes the intersection of disability and age‐related marginalization. It argues that addressing exclusion in agribusiness requires capability‐enhancing approaches, including inclusive infrastructure, disability‐responsive financing, adapted extension services, and sustained public awareness. Such measures are vital for promoting social equity and strengthening national agricultural productivity while advancing sustainable development goals.

## 1. Introduction

Globally, persons with disabilities (PWDs) face persistent barriers in accessing employment opportunities, including the agribusiness sector [1]. Within this group, youth with disabilities (YPWDs) experience layered disadvantage, often excluded both because of their age and their disability status. The definition of “youth” varies across institutions. The United Nations Educational, Scientific, and Cultural Organization [2] defines youth as individuals aged 15–24. Similarly, the United Nations [3] and the World Health Organization (WHO) consider youth to encompass those aged 15–24 [4], whereas the International Labor Organization [5] adopts the same age range. In contrast, Ghana and the African Union (AU) define youth as individuals aged 15–35 [6, 7]. This study adopts Ghana and the AU′s definitions, recognizing the extended transitions into adulthood and work common across African contexts, where young people often experience prolonged pathways into stable employment and socioeconomic independence due to limited employment opportunities.

Disengagement from agribusiness among youth is a global phenomenon [8, 9]. In the Global North, barriers often arise from technological mismatches and perception gaps; many young people in countries such as Canada and the United Kingdom regard agriculture as outdated and lacking innovation [9, 10]. In South Asia, youth, particularly women and those from marginalized castes, face restrictive social norms, limited access to finance, and gender‐based discrimination [11]. In sub‐Saharan Africa (SSA), these challenges are even more pronounced. Agriculture is frequently perceived by young people as unprofitable, labor‐intensive, and unstable [8]. Structural barriers include inadequate infrastructure, a lack of modern farming inputs, and climate‐induced production volatility. Additionally, access to credit remains critically limited, with only a small proportion of bank lending in the region directed toward agriculture, disproportionately affecting young entrepreneurs [8, 12]. In South Africa, one of the more developed economies in Africa, young people also contend with insufficient access to agricultural information and practical skills [13, 14].

Evidence from both developing and developed countries suggests that YPWDs often face significant barriers to participation in agriculture and agribusiness when compared with their able‐bodied counterparts [15–16]. For example, in Northern Nigeria, young people with disabilities who work in agriculture reported difficulties due to prejudice, limited access to agricultural resources, and insufficient institutional assistance [16]. Similarly, a study among smallholder farmers in Western Kenya found lower labor market participation among PWDs due to structural and socioeconomic constraints [17]. In the United States, disability‐inclusive agricultural programs have been developed specifically to address barriers that limit the participation and productivity of farmers with disabilities [18]. In Ghana, national agricultural policies and programs, such as Planting for Food and Jobs, have largely targeted able‐bodied youth, implicitly assuming physical capacity and mobility and leaving YPWDs largely absent from program design and implementation.

Evidence on disability inclusion in agriculture in Ghana highlights challenges of limited tailored training, negative community attitudes, and exclusion from agricultural inputs and services [19].

Studies across several countries in SSA, including Ghana [20], Kenya [21], Nigeria [22], and Ethiopia [23], identify additional constraints confronting the agricultural sector. These include inadequate policy support, weak institutional capacity, insufficient training, and limited access to markets. For YPWDs, these barriers intersect with disability‐related constraints, stigma, inaccessible infrastructure, communication barriers, and limited assistive technologies, creating compounded exclusion from productive agribusiness opportunities. In many SSA countries, inclusive practices such as sign language interpretation can enable persons with hearing impairments to participate effectively in vegetable production [24–26]. However, such initiatives remain isolated rather than systemic.

Several national initiatives have sought to promote inclusion. The Harnessing Agricultural Productivity and Prosperity for Youth (HAPPY) initiative, targeting youth in rice, soybean, tomato, and poultry value chains, reports that many beneficiaries are PWDs receiving technical training and out‐grower support [27]. Similarly, the “Ghana Grows” program, led by the Mastercard Foundation, has introduced motivational tours in high schools for the deaf, framing agriculture as a viable and inclusive career path [28]. Despite these promising examples, empirical research specifically examining YPWDs′ engagement or nonengagement in agribusiness in Ghana remains limited. This gap constrains policymakers and development actors from designing targeted, evidence‐based interventions.

This study therefore asks two central questions: (i) Are YPWDs engaged in agribusiness in Ghana? (ii) What are their reasons for nonengagement? By combining global and national perspectives, applying a structured definition of youth, and integrating disability inclusion theory, this study moves beyond simple participation counts to interrogate how disability, age, and structural inequities intersect to shape agricultural opportunities for YPWDs. In doing so, it seeks to generate insights that inform inclusive policy and practice that foster the engagement of YPWDs in agribusiness.

## 2. Theoretical Foundation: Inclusive Capability Theory (ICT)

This study is anchored in ICT, a framework evolving from Amartya Sen′s capabilities approach and the principles of disability studies. The theory centers on an individual′s ability to do and become what they value, not merely as outcomes, but as genuine freedoms and opportunities [29, 30]. For YPWDs in agriculture, this framework emphasizes not only their presence in agribusiness, but also their capacity to participate meaningfully, productively, and sustainably. This lens shifts attention from counting participation to examining whether engagement genuinely expands choice, dignity, and long‐term livelihood security.

ICT highlights two intertwined components: capabilities and conversion structures. Capabilities reflect aspirations, skills, and potential, what YPWDs could achieve under equitable conditions. Conversion factors encompass personal (e.g., disability), social (e.g., stigma and cultural norms), and environmental (e.g., infrastructure and land access) conditions that influence whether potential becomes action [30, 31]. For instance, a deaf youth may have the technical ability to manage greenhouse production, yet without accessible training, sign‐language interpretation, and supportive institutional policies, this capability remains unrealized. Similarly, youth‐friendly financial instruments can convert entrepreneurial interest into viable agribusiness ventures. Within this framework, exclusion is interpreted not as individual failing, but as a structural failure to enable capabilities to become real opportunities [32]. Inclusion, therefore, is framed as empowerment and equity rather than charity or accommodation.

A common critique of the framework concerns its abstractness and the difficulty of operationalization [33, 34]. Unlike instrumental models such as social capital frameworks, ICT offers limited prescriptive guidance on program design. However, its analytical depth makes it particularly useful for examining systemic exclusion and power relations. In this study, the framework is applied primarily as an interpretive lens rather than a rigid evaluative model, enabling attention to lived experience, institutional practices, and broader social arrangements. By examining how age, disability, education, finance, policy, and social norms intersect, the study illuminates how capabilities, such as managing an agribusiness, are either realized or constrained. This theoretical anchor enhances conceptual clarity while guiding interpretation toward transformative and justice‐oriented policy solutions. Throughout the analysis, ICT demonstrates that YPWDs may possess interest, skills, and motivation, yet remain constrained by inaccessible markets, institutional neglect, and persistent stigma, ultimately limiting their agency and freedom of choice [35–37].

## 3. Methods

### 3.1. Study Setting

This study is part of a broader study that was conducted across the three main agroecological zones of Ghana: southern, middle, and northern zones. These zones represent diverse agricultural systems, climatic conditions, and sociocultural contexts that influence the participation of PWDs in agribusiness activities. In 2021, Ghana had an estimated population of 30.8 million, and an estimated 2.1 million of its population older than 5 years had difficulties in performing activities, such as seeing, walking, hearing, and self‐care [38, 39]. In addition, 32% of Ghana′s population aged 15 years and older were engaged in agricultural‐related activities in 2021 [40].

### 3.2. Study Design and Sampling

The broader study adopted a mixed‐methods design combining quantitative and qualitative approaches [41]. The authors were actively involved in the conceptualization, design, and implementation of the broader mixed‐methods study. While the broader study examined multiple dimensions of the livelihoods and inclusion of PWDs across Ghana′s three agroecological zones, the present paper draws on a subset of the data and focuses specifically on youth with disabilities (YPWDs), their engagement in agribusiness, and reasons for nonengagement. The study design was chosen to triangulate patterns observed in survey data with rich, contextual explanations from the qualitative data (in‐depth interviews [IDIs] and focus group discussions [FGDs]), thereby providing comprehensive insights into the experiences, challenges, and opportunities of YPWDs in agriculture. The study formed part of a broader needs‐assessment intended to inform interventions for YPWDs within the agricultural value chain under the HAPPY program, while retaining analytical independence for academic reporting. The broader study covered several issues, including economic information, interests and specializations, challenges and support, accessibility and inclusion, and agricultural techniques and practices. However, this study focused on the economic information of PWDs, specifically their engagement in agribusiness and reasons for nonengagement in agribusiness.

#### 3.2.1. Quantitative Sampling

Twelve districts were purposively selected from six regions to reflect the ecological spread: Greater Accra, Volta, Ashanti, Bono, Upper East, and Northern regions. Within each district, three communities were selected in consultation with local authorities and disability associations to ensure inclusion of areas with known populations of PWDs. This approach helped capture experiences across both rural and periurban contexts.

To determine the sample size, for the broader study population of PWDs irrespective of age, Taro Yamane′s (1967) formula was applied using a 5% margin of error, yielding a minimum sample size of 384 respondents. The sample was distributed evenly across the 12 districts (32 respondents per district). Snowball sampling was employed to reach respondents, facilitated through local networks and associations under the Ghana Federation of PWDs [42]. This approach was particularly useful in contexts where disability is stigmatized and individuals are not readily identifiable in public records. Efforts were made to ensure the participation of youth and women with disabilities.

#### 3.2.2. Qualitative Sampling

Purposive sampling was used in the broader study to identify participants for IDIs and FGDs [43]. Participants included PWDs, caregivers, community leaders, officials from ministries, departments, and agencies (MDAs), NGO representatives, and value‐chain actors. Selection emphasized maximum variation (age, disability type, gender, and geographical location) to ensure diversity of experience. Gender‐segregated FGDs (five to seven participants each) were conducted to encourage open discussion. In total, 11 national‐level IDIs, 36 district‐level IDIs, and 24 community‐level FGDs were conducted. The findings of this study focused on the accounts of YPWDs.

### 3.3. Types and Sources of Data

Data sources included both primary and secondary materials. Primary data were collected through surveys, FGDs, and IDIs, whereas secondary data were obtained from desk reviews, literature on disability and agribusiness, and relevant policy frameworks. Integrating multiple data sources allowed cross‐validation of findings and supported deeper interpretation of structural and experiential barriers affecting YPWD engagement.

### 3.4. Data Collection

#### 3.4.1. Quantitative Data Collection

Structured surveys were administered using electronic data collection tools (KoboCollect) to ensure efficiency and real‐time data capture. These tools minimized human error and facilitated smooth transfer to analysis software. The questionnaire collected data on demographic characteristics, types of disabilities, participation in agricultural activities, access to services, and perceived barriers and enablers. Enumerators were trained intensively on disability‐sensitive data collection, inclusive language, ethics, and use of assistive devices. They were paired with interpreters when necessary to support communication with hearing or speech‐impaired respondents. Where needed, interpreters and assistive tools were provided to facilitate participation of people with hearing, speech, or visual impairments [44, 45].

#### 3.4.2. Qualitative Data Collection

IDIs and FGDs were conducted using semistructured interview guides that were developed based on themes derived from the scoping literature review. Key themes included accessibility to land and finance, stigma and discrimination, training, knowledge transfer, and institutional support. Interviews were conducted in local languages where necessary and translated into English for transcription. All sessions were audio‐recorded with participants′ consent. Reflexive field notes were taken to capture nonverbal dynamics, contextual factors, and researcher impressions. Separate FGDs were conducted for male and female participants to ensure safe and open dialogue [43, 46].

### 3.5. Data Analyses

#### 3.5.1. Quantitative Analysis

Quantitative data were cleaned, coded, and analyzed in SPSS. Descriptive statistics summarized frequencies and percentages. Cross‐tabulations explored relationships between variables such as age, sex, disability type, and participation in agribusiness. Chi‐square tests were applied where appropriate to identify statistically significant associations. These analyses helped identify patterns across demographic groups. The broader study surveyed 472 respondents with disabilities across all age categories, exceeding the minimum sample size requirement of 384 respondents. Since this study focused on YPWDs, the data analyzed were restricted to 169 respondents aged 18–35.

#### 3.5.2. Qualitative Analysis

Qualitative data were transcribed verbatim and analyzed using NVivo software. Two researchers independently coded a subset of transcripts and discussed discrepancies to enhance analytic reliability. Thematic analysis was employed to identify recurring patterns and themes that emerged from the narratives of YPWDs and stakeholders [46]. Codes were developed both deductively, based on research questions, and inductively, based on emerging data. The final themes reflected lived experiences, structural barriers, and cultural contexts influencing YPWDs′ engagement in agribusiness. Triangulation between qualitative and quantitative findings was applied to validate and enrich interpretations, ensuring both depth and breadth of analysis [47].

### 3.6. Ethical Clearance

The study adhered to ethical standards protecting the rights and welfare of participants, particularly considering the vulnerabilities associated with disability [44, 45]. Ethical approval was obtained from the Ethics Committee of the University of Environment and Sustainable Development prior to the commencement of data collection on September 6, 2024. Informed consent was sought from all participants, including simplified consent procedures for those with intellectual or communication disabilities. Confidentiality was maintained by anonymizing data and limiting access to transcripts and recordings. Participants were also compensated for transport expenses, and all interactions followed strict protocols for respectful and culturally sensitive engagement. To ensure responsible representation and inclusion, ethical guidelines and participant engagement protocols were developed and implemented throughout the study. Interviewers and facilitators received training on disability‐sensitive research practices, including respectful communication, informed consent procedures, and the identification of participant distress or discomfort. When necessary, interviews or discussions were paused or terminated to safeguard participants′ well‐being.

### 3.7. Transparency in Use of AI Tools

In accordance with journal guidelines, portions of language editing and formatting support were provided using AI‐assisted writing tools. These tools were not employed for data generation, analysis, or interpretation. All substantive judgments, analytical decisions, and conclusions were made by the authors.

## 4. Results

### 4.1. Sociodemographic Characteristics of Respondents

The study involved 169 YPWDs drawn from various regions across Ghana (see Table 1). In terms of sex, more than half of respondents (57.4%) were females, whereas 42.6% were males. Three‐fifths of respondents (60.9%) were aged 27 years and older, and a majority of respondents (68.0%) had formal education.

**Table 1:** Sociodemographic characteristics of YPWDs.

Variable	Frequency	Percentage
Sex		
Male	72	42.6
Female	97	57.4
Age		
18–26 years	66	39.1
27–35 years	103	60.9
Education		
No formal education	54	32.0
Less than senior high school	84	49.7
Senior high school and higher	31	18.3
Marital status		
Never married	111	65.7
Currently married	50	29.6
Formerly married	8	4.7
Religion		
No religion	8	4.7
Christianity	130	76.9
Other religion	31	18.4
Ecological zone of regions		
Southern zone	64	37.9
Middle belt	39	23.0
Northern zone	66	39.1
Currently working		
Yes	53	31.4
No	116	68.6
Engaged in agribusiness (*n* = 53)		
Yes	28	52.8
No	25	47.2
Total	169	100.0

Additionally, marital status data showed that most respondents (65.7%) were never married, whereas about 3 out of 10 respondents (29.6%) were currently married. Most respondents were Christians (76.9%). Geographically, most respondents resided in the northern zone (39.1%), followed by the southern zone (37.9%) and the middle belt (23.0%), reflecting a broad yet uneven geographic spread as indicated in Table 1. Furthermore, only 31.4% of respondents were currently employed.

### 4.2. Engagement in Agribusiness

Among the 53 YPWDs who were currently employed, a little over half (52.8%, *n* = 28) were engaged in agribusiness. YPWDs′ engagement in agribusiness is shaped by intersecting personal, social, and structural barriers. As shown in Table 2, females accounted for a greater share of YPWDs engaged in agribusiness than males (57.1%, *n* = 16 vs. 42.9%, *n* = 12). In terms of age, the majority of respondents (75.0, *n* = 21) were aged 27 years and older.

**Table 2:** YPWDs working in agribusiness sector by sex, age, education, marital status, and religion.

Variable	Frequency	Percentage
Sex		
Male	12	42.9
Female	16	57.1
Age		
18–26 years	7	25.0
27–35 years	21	75.0
Education		
No formal education	9	32.2
Less than senior high school	13	46.4
Senior high school and higher	6	21.4
Marital status		
Never married	14	50.0
Currently married	12	42.9
Formerly married	2	7.1
Religion		
No religion	1	3.6
Christianity	26	92.8
Other religion	1	3.6
Ecological zone of regions		
Southern zone	12	42.9
Middle belt	12	42.9
Northern zone	4	14.2
Total	28	100.0

Educational attainment among YPWDs in agribusiness revealed that 32.2% (*n* = 9) had no formal education, whereas 21.4% (*n* = 6) had senior high school and higher education. Also, 46.4% (*n* = 13) of respondents had less than senior high school education.

With respect to marital status, half of the respondents (50.0%, *n* = 14) were never married. About 43% (*n* = 12) of respondents were currently married, whereas 7.1% (*n* = 2) were formerly married. The majority of YPWDs in agribusiness were Christians (92.8%, *n* = 26), while most YPWDs in agribusiness were located in the Southern zone (42.9%, *n* = 12) and Middle belt (42.9%, *n* = 12), respectively.

### 4.3. Reasons for Nonengagement in Agribusiness

Despite increasing efforts to promote inclusive participation in agriculture, many YPWDs remain disengaged from agribusiness. Through the IDIs and FGDs, participants highlighted a complex set of barriers that hinder their involvement. Seven recurring themes emerged and are discussed below with supporting narratives from the field.

#### 4.3.1. Difficult to Access Agricultural Work

Many YPWDs expressed that agricultural spaces are physically inaccessible. Their impairments make it challenging to participate in demanding fieldwork, especially in areas without adapted tools or support systems.

“I tried joining a group on a cassava farm, but the place was too far and hilly. I use crutches and cannot walk that long. I just gave up and stayed home. If there were easier jobs or transport, I would have continued.” (Female, 27 years, visually impaired).

“Most farms are outside town and require long hours of standing or bending. For someone like me who uses a wheelchair, it′s just not practical. No one even considers building something like an accessible garden.” (Male, 22 years, wheelchair user).

#### 4.3.2. Discrimination or Stigma

Approximately 4 in 10 respondents identified discrimination or stigma as a barrier to their engagement in agribusiness. Stigmatizing attitudes and discriminatory beliefs were reported as key deterrents, with some youth noting that they were openly discouraged or excluded from agricultural activities because of their disability.

“When I expressed my desire to learn poultry farming, the trainer laughed dismissively and said, ‘You? Even the chicken will feel sorry for you.’ That hurt deeply, revealing a pervasive belief that people like me cannot achieve anything, especially in farming.” (Female, 19 years, hearing impaired).

“Many believe that we are weak and should avoid the sun or handling tools. Even when I express a desire to try, they dismiss me, saying, ‘This is not for disabled people.’ Such attitudes discourage many of us from pursuing our ambitions.” (Male, 33 years, upper limb amputee).

#### 4.3.3. No Land Available for Farming

Land access remains a structural barrier for many YPWDs, especially those without family support or community ties. Customary land tenure systems often ignore the needs of disabled youth.

“My family owns land, but my uncle insists I should leave it for my brothers, claiming I would not use it properly. He told me that farming is not for someone like me and suggested I should focus on begging instead. This dismissive attitude deeply frustrates me.” (Male, 25 years, lower limb amputee).

“Even if I had the strength to farm, I face a major barrier: I have no land. Landlords are often unwilling to lease to people like us, believing we will waste it or fail. This prejudice prevents many from pursuing their farming dreams.” (Female, 31 years, visually impaired).

#### 4.3.4. No Money or Access to Loans

A lack of financial resources and challenges in accessing credit schemes specifically designed for YPWDs were frequently cited as significant barriers to both starting and sustaining agricultural business ventures. These obstacles hinder potential growth and limit opportunities in the sector.

“I aspired to raise goats, but the little money I had was consumed by hospital bills. When I applied for a loan to get started, I was told I did not qualify due to a lack of a guarantor and land. This setback felt discouraging.” (Male, 29 years, speech impaired).

“Every business begins with capital, but who provides it for us? They tell us to pursue farming, yet banks require security, and NGOs expect us to already have a venture underway. It leaves me wondering: where am I supposed to start?” (Female, 24 years, epileptic).

#### 4.3.5. No Training

YPWDs have expressed a significant lack of access to training opportunities in agribusiness. This is particularly true for programs that are inclusive and consider their unique learning styles and mobility limitations, further hindering their ability to enter and succeed in the agricultural sector.

“They organize training programs, but we do not hear about them or they do not think of us. One time, they had a fish farming class in a place I could not even enter because of steps.” (Female, 20 years, wheelchair user).

“If we had access to training, perhaps we would be more willing to try something new. However, most training programs are designed for able‐bodied individuals, leaving us completely excluded. This lack of opportunity makes it difficult to pursue our ambitions in agribusiness.” (Male, 35 years, hearing impaired).

#### 4.3.6. Not Enough Knowledge About Farming

Some respondents stated they were simply unaware of what agribusiness entails or how to get started. Limited exposure to agricultural education in school or their communities contributed to this:

“I grew up in town, so I have no knowledge of farming at all. No one ever taught me about it, and now I feel completely lost about where to begin. If I had some guidance, I might be willing to give it a try and explore this opportunity.” (Male, 18 years, clutches user).

“People often encourage us to go into agriculture, but no one explains what kind of farming we can pursue or how to adapt it to our disabilities. There′s so much we do not know, and the lack of clarity is overwhelming.” (Female, 21 years, hearing impaired).

#### 4.3.7. Not Interested in Agriculture

Finally, some YPWDs simply did not view agriculture as appealing. Their disinterest was influenced by prior negative perceptions, a desire for urban or tech‐based jobs, or a belief that agriculture is outdated.

“I just do not like farming; it′s hard work, and I′ve never had any interest in it. I would much rather learn computer skills or open a shop. To me, farming feels like something for older generations, not for someone like me.” (Male, 19 years, visually impaired).

“Even if they offered me land and tools, I would not want to go into farming. My goal is to work in an office. We should have the freedom to choose our own paths and not be forced into farming just because we are disabled.” (Female, 23 years, speech impaired).

## 5. Discussion

This study sought to understand whether YPWDs in Ghana are engaged in agribusiness and to identify the factors contributing to their nonengagement. Through a mixed‐methods approach, the study uncovered a nuanced landscape of marginalization, resilience, and structural limitations. Quantitative data revealed that 52.8% of employed YPWDs are engaged in agribusiness. Agribusiness participation is largely driven by older female youth who have attained limited formal education, particularly in rural settings. These findings resonate with broader global patterns where agricultural engagement is often constrained, especially among marginalized populations [8, 12]. They also align with emerging agribusiness research indicating that entry into agricultural value chains increasingly requires skills, networks, capital, and technology, all of which tend to exclude youth with disabilities.

Regionally, youth in SSA frequently migrate away from agriculture due to limited income prospects, poor working conditions, and weak integration into value chains [8, 48]. Many young adults do not see agriculture as a viable long‐term livelihood because it remains labor‐intensive with limited returns [49].

Participants identified multiple intersecting barriers reflecting systemic exclusion. Physical inaccessibility and stigma emerged as persistent obstacles. Farms and production sites were often difficult to navigate, and discriminatory attitudes undermined confidence and participation. These findings are consistent with global evidence showing stigma as a powerful exclusionary mechanism in agricultural livelihoods [50, 1]. Viewed through ICT, such barriers weaken substantive freedoms, leading to “choices” that are in reality constrained by structural denial rather than preference.

Access to land and finance represented another critical barrier. Customary tenure systems and discriminatory credit practices limited access for YPWDs [51–53]. Without land, capital, or assets, YPWDs are structurally prevented from entering even low‐level segments of agricultural value chains. Exclusion therefore stems not from personal deficits but from institutional arrangements that fail to recognize disability‐inclusive access as part of agrarian justice.

Lack of accessible training and knowledge further constrained participation. At the national level in Ghana, it is noteworthy that participation in agricultural programs and training opportunities is not determined by educational qualifications. Agricultural programs rarely adapt curricula, spaces, or communication strategies to disability needs. This gap prevents capability activation [15, 31]. Training opportunities, including growing crops or raising animals, using machinery or tools for farming, adding value to products and selling products, must therefore move beyond enrollment numbers toward inclusive design, adaptive technologies, and ongoing mentorship.

Some YPWDs expressed disinterest in agriculture, instead preferring urban‐based or technology‐driven livelihoods. Consistent with global trends, these preferences reflect perceived prospects for autonomy and economic returns [54, 55]. ICT helps interpret this as constrained choice: youth gravitate toward sectors where structural conditions appear more enabling, not necessarily because agriculture lacks value.

Overall, the study shows that participation is limited not because YPWDs lack ability or motivation, but because agricultural systems remain structurally exclusionary. By foregrounding disability within youth‐and‐agriculture debates, this study advances agribusiness scholarship that has historically treated both categories independently [56–58].

Although some YPWDs successfully participate through family networks, these cases are exceptions. Inclusion requires expansion of enabling “conversion structures”: accessible credit, inclusive extension services, adaptive training, and equitable land access [8, 59]. Disability must shift from being peripheral to becoming a core‐organizing principle in agricultural programming.

In sum, inclusion in agriculture extends beyond participation rates to encompass enabling freedoms, including equitable access to markets, resources, education, community services, and safety nets against disability‐related exclusion. This study demonstrates that disability‐inclusive agribusiness should be pursued not as charity, but as a question of capability, dignity, and justice. These findings have implications beyond Ghana. Across many countries in Africa and the Global South, young persons with disabilities continue to experience exclusion from productive sectors despite policy commitments to inclusive development. The barriers identified in this study, including limited access to land, finance, training, and supportive institutions, mirror challenges documented elsewhere in low‐ and middle‐income countries. More broadly, disability‐inclusive agricultural development aligns with regional and global commitments, including the AU Agenda 2063 [60], the United Nations Convention on the Rights of Persons with Disabilities [44], and the Sustainable Development Goals [61]. These frameworks call for equitable participation in economic and social life, underscoring the need to move beyond welfare‐oriented approaches toward rights‐based and capability‐enhancing interventions.

## 6. Limitations and Strengths

This study offers important insights into YPWDs engagement in agribusiness in Ghana; however, several limitations warrant consideration. Snowball sampling, although effective in reaching hidden populations, may introduce selection bias, as respondents are often socially connected. As a result, the most socially isolated youth, who may also be the most capability‐deprived, may be underrepresented. A key limitation of this study is the small number of YPWDs included. Future studies should employ larger, nationally representative respondents to enhance understanding of their participation in agribusiness.

Language and communication challenges may also have affected interpretation, despite the use of interpreters and trained enumerators. Social desirability bias may have influenced responses, particularly in conversations about stigma or dependence.

Logistical constraints across three ecological zones and 12 districts also posed challenges, and variations in enumerator experience may have introduced inconsistencies. Future studies could benefit from longitudinal and randomized sampling strategies to enhance representativeness.

Despite these constraints, the mixed‐methods approach strengthened validity through triangulation of survey and narrative data. Gender‐segregated FGDs enhanced comfort and openness. By situating findings within ICT, the study provides a theoretically grounded account of how structural conditions shape participation, offering a solid platform for policy and programmatic intervention.

Although the study is situated within Ghana, the issues identified are relevant to many low‐ and middle‐income countries where young persons with disabilities face similar structural barriers to economic participation. The findings therefore offer insights that may inform disability‐inclusive agricultural programming and occupational participation strategies in comparable contexts, while acknowledging that institutional and cultural differences may affect transferability.

## 7. Conclusion

This study explored engagement of YPWDs in agribusiness in Ghana and identified reasons for nonparticipation. YPWDs′ engagement in agribusiness was 52.8%, and it was shaped by intersecting personal, social, and structural barriers. Only a small proportion of YPWDs, older female youth with limited formal education in rural areas, were involved in agriculture, often under precarious and informal arrangements. Seven major constraints: limited access to work opportunities, stigma, lack of land, lack of capital, absence of training, inadequate knowledge, and disinterest represent forms of capability deprivation. Opportunities exist in principle, but enabling environments are weak.

Applying ICT, the study shows that exclusion is rooted in institutional and structural shortcomings rather than individual incapacity. A rights‐based approach to disability inclusion is therefore essential. Youth and disability must be addressed together rather than in parallel policy silos. This work contributes to emerging scholarship on inclusive agribusiness by demonstrating how capability expansion can inform more just, dignified, and equitable agricultural systems. Future research should investigate scalable disability‐inclusive models, including digital agriculture, cooperatives, and adaptive technologies, ideally through longitudinal designs.

The findings also have important implications for policy and practice. National agricultural and youth strategies should explicitly recognize YPWDs as a priority demographic through disability‐responsive targets, reporting indicators, and outreach strategies. Constraints relating to land and finance require targeted interventions, including accessible microfinance, grant schemes, cooperative land models, and safeguards against discriminatory lending practices. Agricultural training and extension services should be redesigned to accommodate diverse disability needs through accessible communication formats, inclusive training environments, and adaptive technologies. Addressing stigma through community engagement and public awareness initiatives remains equally important. More broadly, achieving meaningful inclusion will require coordinated action across the agriculture, youth, education, social protection, and local governance sectors. Such integrated approaches are necessary to unlock the potential of YPWDs as contributors to sustainable food systems, rural transformation, and inclusive development in Ghana and comparable contexts across Africa and the Global South.

## Funding

No funding was received for this manuscript.

## Ethics Statement

Ethical approval for this study was granted by the University Ethics Committee (UEC) of the University of Environment and Sustainable Development, Certificate Reference No. UESD/UEC/ECC/24/0003, on September 6, 2024, prior to data collection. Written informed consent was obtained from all participants before participation. All study procedures were conducted in accordance with the ethical principles of the Declaration of Helsinki and relevant national and institutional guidelines for research involving human participants, including youth with disabilities.

## Consent

Informed consent was obtained from all participants prior to data collection. Participants were assured of the voluntary nature of their participation, their right to withdraw at any time, and the confidentiality of their responses. All identifying information was anonymized or altered during transcription and analysis to ensure participant privacy.

## Conflicts of Interest

The authors declare no conflicts of interest.

## Data Availability

Due to the sensitive nature of the qualitative and quantitative data involving YPWDs, the datasets generated and analyzed during this study are not publicly available in order to protect participant confidentiality, safety, and anonymity. De‐identified excerpts or aggregate datasets can be made available upon reasonable request to the corresponding author.
